# A rare case of primary cardiac sarcoma with both intracranial metastasis and embolic infarction in a pediatric patient

**DOI:** 10.1007/s00381-026-07434-2

**Published:** 2026-08-19

**Authors:** Mason T. Stoltzfus, Jinpyo Hong, Thaddeus Harbaugh, Jason Sherman, Khoa D. Pham, Michael McDowell

**Affiliations:** 1 Department of Neurosurgery, Penn State Health Milton S. Hershey Medical Center, 500 University Drive, Hershey, PA 17033 USA; 2 New Jersey Medical School, Newark, NJ USA

**Keywords:** Cardiac sarcoma, CNS metastasis, Postoperative outcomes

## Abstract

An 18-year-old Hispanic male with an unremarkable medical history presented with a 1-month history of progressive, intermittent headaches, nausea, and diplopia. Upon seeking care, a head CT revealed a solid right frontal lesion and a large, complex cystic parieto-occipital lesion exerting significant mass effect on surrounding structures. The patient initially underwent a left parietal craniotomy for tumor resection, followed by resolution of his neurological symptoms. However, surveillance imaging 1 month later identified a known left atrial cardiac mass and new intracranial embolic infarcts, alongside a 6-cm recurrence at the primary resection site. The patient subsequently underwent a sternotomy for resection of the primary cardiac sarcoma and reconstruction of the left atrium. Shortly after, a second craniotomy was required for resection due to rapid cystic recurrence of the brain metastasis causing midline shift and new focal neurological deficits. Following successful gross-total re-resection, the patient underwent hypofractionated Gamma Knife radiosurgery. He is also undergoing chemotherapy. Six-week follow-up imaging confirmed a positive response to treatments, showing no new metastatic growth and a reduction in the size of existing lesions. This case highlights the aggressive nature of primary cardiac sarcoma and the necessity of multimodal, repeat surgical intervention for CNS involvement.

## Introduction

Primary cardiac tumors are rare, with an estimated prevalence of about 0.0017% to 0.028% based on autopsy studies and large registries [1]. Most of these tumors are benign, the most common being myxomas [2]. A small subset of primary cardiac tumors are malignant, the most common of these being sarcomas [3]. Even though they are rare, malignant primary cardiac tumors are associated with poor outcomes, with population-based studies reporting a 5-year survival rate of 10–15% [4].

Although primary sarcomas often metastasize to the lungs, bone, and liver, cardiac sarcomas show a notably greater tendency for central nervous system (CNS) involvement compared to extracardiac sarcomas [4, 5]. CNS manifestations from primary cardiac tumors typically arise by one of two mechanisms. Benign myxomas tend to cause embolic infarcts when tumor fragments or thrombi enter the cerebral circulation [2, 7]. In contrast, malignant tumors can disseminate hematogenously and form parenchymal brain metastases, typically presenting with seizures, focal neurologic deficits, or symptoms of increased intracranial pressure. Malignant tumors also commonly present as hemorrhagic lesions on imaging [5, 7, 8]. The current literature on brain metastases from cardiac sarcoma largely consists of case reports covering rhabdomyosarcoma, angiosarcoma, and high-grade pleomorphic or myxofibrosarcoma variants, commonly in middle-aged adult patients presenting with new neurologic symptoms [9–16].

We report an 18-year-old male with a large left atrial cardiac mass and multiple intracranial lesions presenting with headaches, diplopia, and visual field loss, including a cystic parieto-occipital metastasis and a contralateral deep white matter lesion. Reports of adolescents presenting with multiple brain lesions from a primary cardiac sarcoma are uncommon, and the published literature on cardiac sarcoma with multifocal CNS involvement remains limited [12–15]. This case highlights the diagnostic and therapeutic challenges of managing a large left atrial cardiac tumor alongside rapidly progressive intracranial disease in a young patient.

## Case presentation

**Fig. 1:**
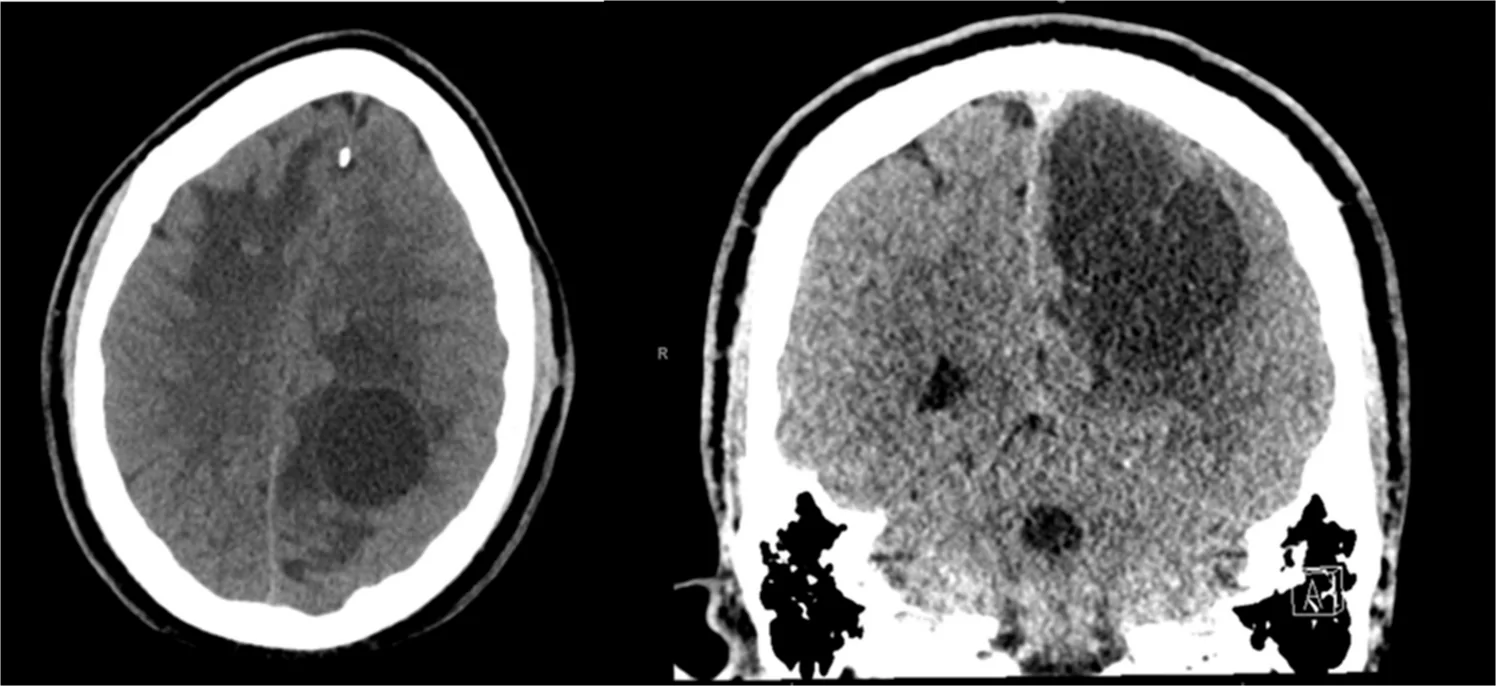
Patient’s initial CT head without contrast with a heterogenous parieto-occipital lesion with a 3.8-cm cystic component and a 3-cm solid component and a hyperdense 1.1-cm solid right frontal centrum semiovale lesion with significant surrounding vasogenic edema best demonstrated in the left image

An 18-year-old Hispanic male with no significant medical, social, or family history presented with a month of intermittent headaches. During the week prior to presenting, he also developed nausea, diplopia, and worsening of his headaches. CT scan of the head revealed a hyperdense 1.1-cm solid right frontal centrum semiovale lesion with significant surrounding vasogenic edema and a 3.8-cm heterogenous parieto-occipital lesion with surrounding vasogenic edema and mass effect on the atrium of the left lateral ventricle as shown in Fig. 1.

CT scans of the chest, abdomen, and pelvis revealed a 5.9-cm low-density, enhancing left atrial mass and benign-appearing calcified nodule of the right upper lung lobe.

**Fig. 2:**
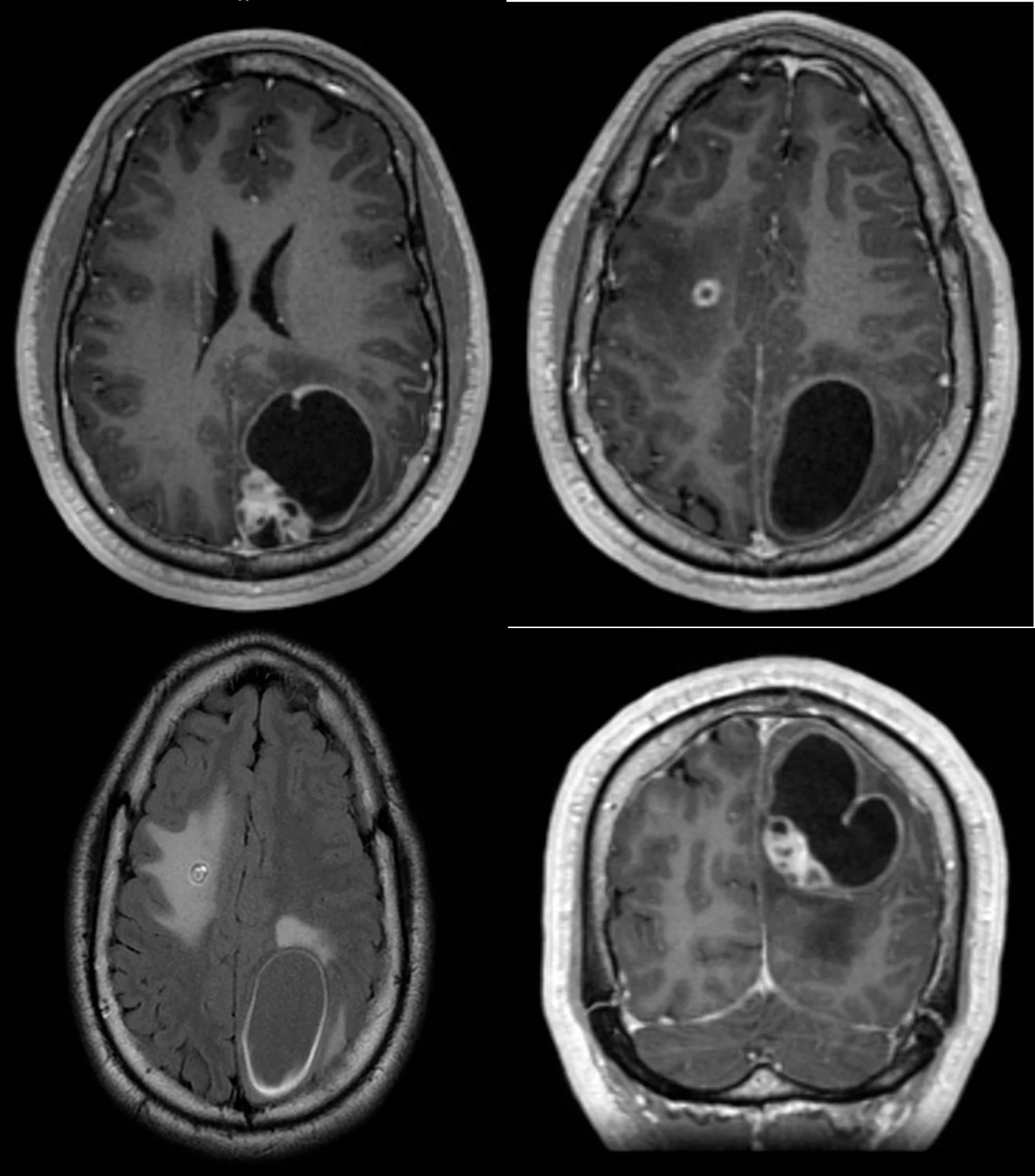
Patient’s initial MRI of the brain (bottom left: T2 FLAIR sequence, all other images T1 post-contrast sequences) further characterizing the left occipito-parietal lesion as mixed cystic and nodular with avid contrast enhancement and mild surrounding vasogenic edema with the ring-enhancing right centrum semiovale mass

Upon admission to the neurosurgery service, the patient was noted to be hypertensive with systolics in the 160 s, had mild headache, and had diplopia with a right inferotemporal quadrantanopia, but was otherwise neurologically intact. An MRI scan of the brain further characterized the left parietal as having enhancing nodular and cystic components, with mild vasogenic edema surrounding the entirety of the lesion as shown in Fig. 2. The primary differential diagnosis at this time was neurocysticercosis or cystic metastatic disease. MRI scan of the pan-spine revealed several vertebral body hemangiomas but no additional lesions with malignant characteristics as shown in Fig. 2.

**Fig. 3:**
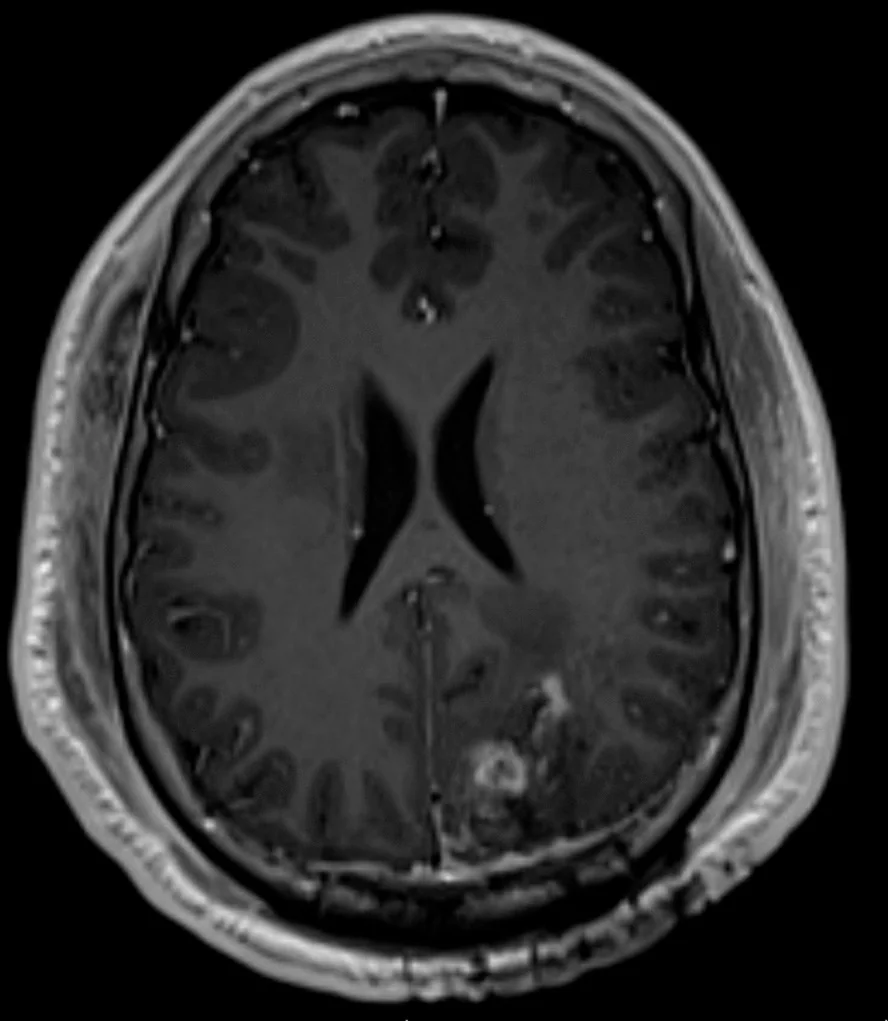
MRI brain T1 post-contrast sequence following resection of the parieto-occipital lesion, revealing gross-total resection of the lesion with some enhancing surgical products visible

The patient underwent a left parietal craniotomy for resection of the parieto-occipital lesion. The patient tolerated the procedure well and experienced complete resolution of his headache and visual deficits. A postoperative MRI scan of the brain demonstrated gross-total resection of the lesion as shown in Fig. 3.

The immunohistochemical stains of CD3, CD20, CD30, Alk1, PAX5, GFAP, S100, CD31, CD34, INI1, pankeratin, actin, EMA, CD45, CD163, and CD68 of the first resection conducted were generally negative except for possible staining of internal control elements.

The morphological findings of the resected tissue revealed a poorly differentiated tumor, composed of cells of variable morphology ranging from cells with ovoid irregular nuclei, distinct nucleoli, and relatively scant cytoplasm to cells with spindle nuclei and somewhat vacuolated cytoplasm entrapped in a densely sclerotic stroma. Mitotic activity was high, with at times multiple mitoses (up to 5) in a single high-power field. The tumor was hemorrhagic, without definite evidence of necrosis, and was accompanied in areas by a marked inflammatory infiltrate with lymphocytes and a high number of histiocytes. ALK1 was negative, and INI1 expression was retained. GFAP and S100 protein stains were negative as well. EMA showed focal faint positivity, while smooth muscle actin was negative. Additional IHC stains showed focal positivity for desmin and cytokeratin OSCAR and negative for CD34, FLI1, and CD31. MDM2 FISH study was positive.

Molecular studies showed amplification of CDK4 and MDM2 via CNA-seq, while BRAF, MSI, NTRK1/2/3, RET, tumor mutational burden, ARID1A, CDKN2A, CDKN2B, EED, ER8B2, EWSR1, EZH2, MAGE-A4, NAB2, RB1, and SMARCA4 were unremarkable. EGFR mutation was not detected in exons 18 through 21, and a sarcoma comprehensive NGS fusion panel was negative.

**Fig. 4:**
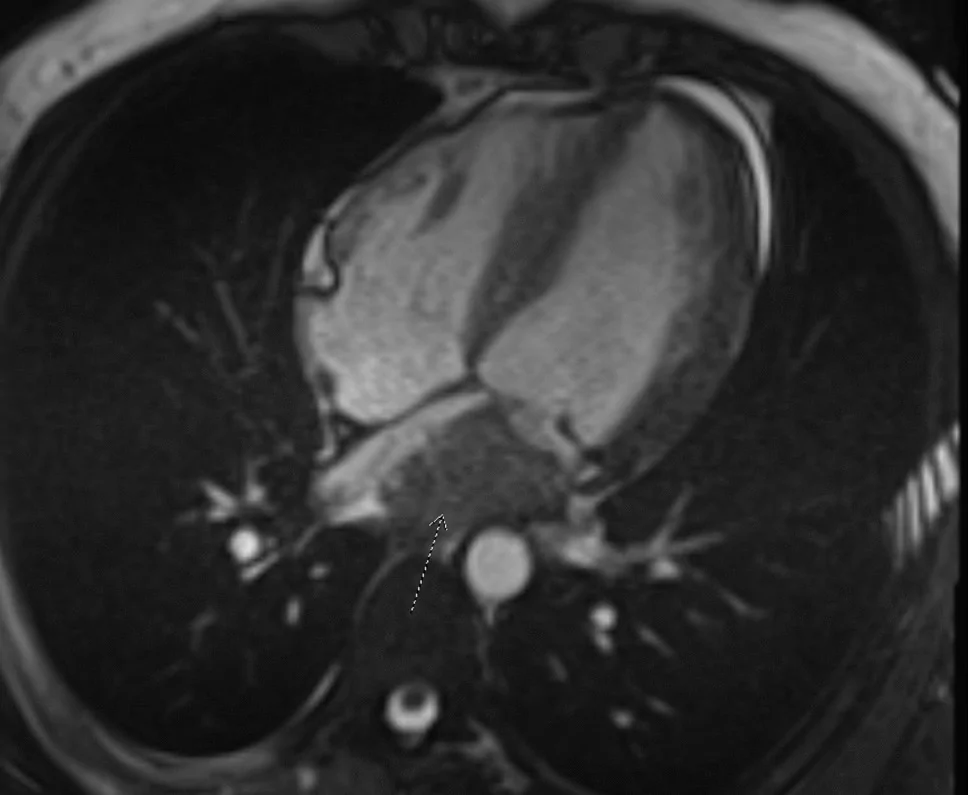
Preoperative cardiac MRI T2 sequence demonstrates a hypointense lesion filling the majority of the left atrium as well as apical pericardial effusion

Cardiology consultants conducted further workup of his left atrial lesion with a contrast-enhanced cardiac MRI scan. The lesion was well-circumscribed with broad-based attachment to the posterior and lateral walls of the left atrium, occupying 70–75% of the atrial volume as shown in Fig. 4. The lesion did not infiltrate the left atrial wall or surrounding structures. The distal segment of the lesion was noted to slightly traverse the mitral annular plane during systole. The lesion was slightly T1 hyperintense and T2 hyperintense with heterogenous late gadolinium enhancement. Radiographically, the lesion most closely resembled an atrial myxoma or malignancy. Slight anterior pericardial effusion was also present, and systolic function was preserved.

**Fig. 5:**
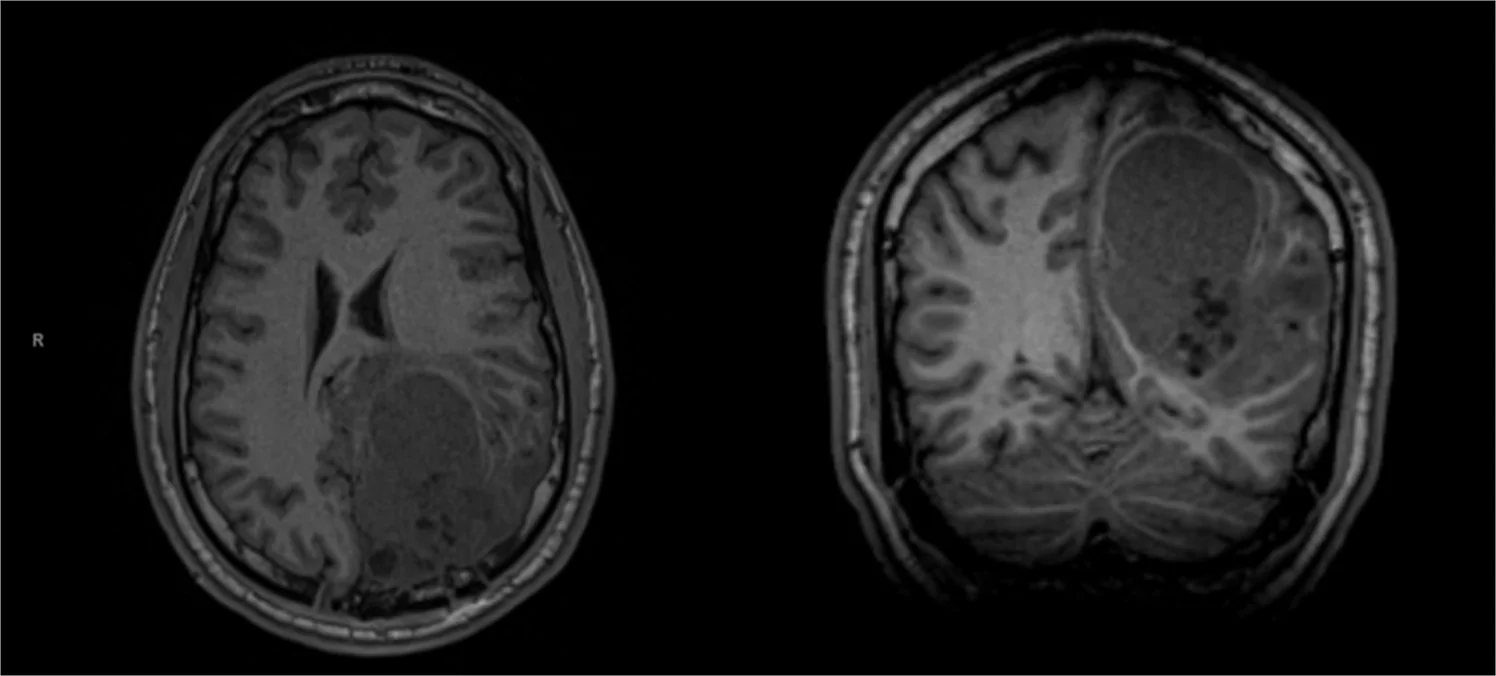
MRI brain T1 post-contrast sequence (with poor timing of contrast administration) revealing recurrence of tumor, demonstrated as a nodulocystic lesion in the left parietal lobe

The patient subsequently underwent scheduled sternotomy for resection of his left atrial cardiac lesion and reconstruction of his left atrium. Two weeks later, the patient presented to a Penn State satellite campus after 3 days of headache and nausea. An MRI brain in Fig. 5 demonstrates an increase in size of the now predominantly cystic left parieto-occipital lesion, now 7.9 cm with 7 mm of midline shift. The right centrum semiovale lesion continued to decrease in size.

**Fig. 6:**
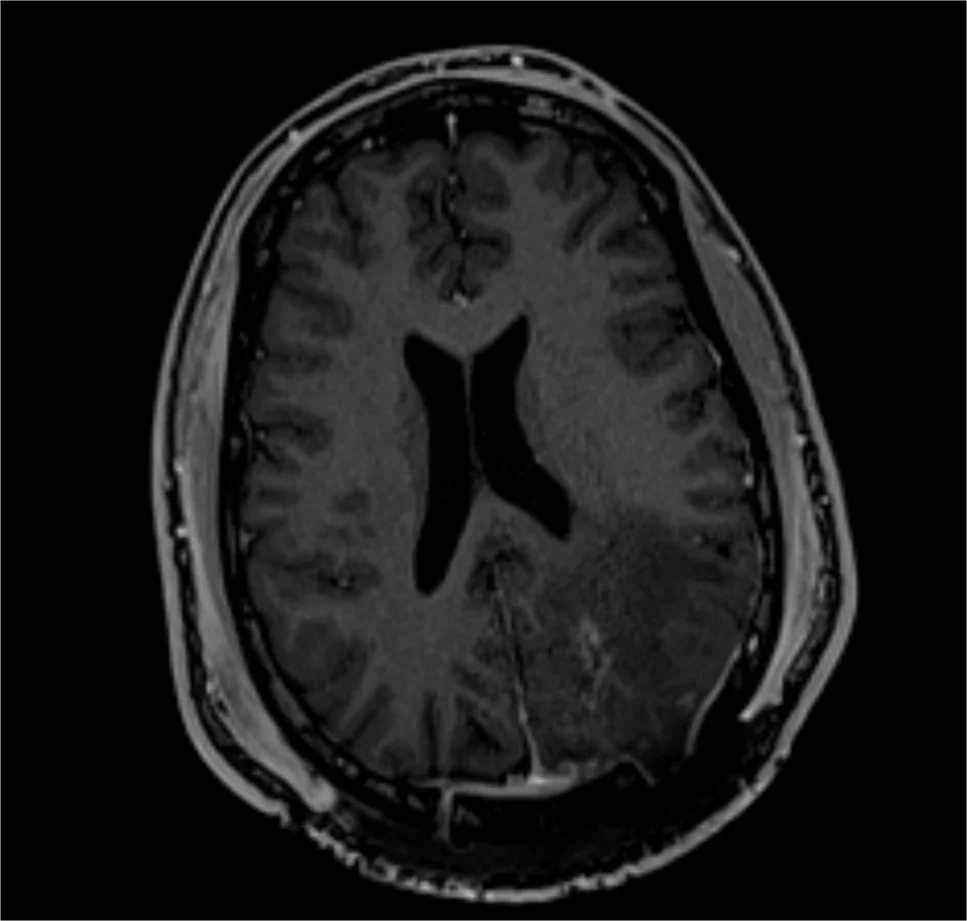
MRI brain T1 post-contrast sequence obtained after re-resection of the lesion, demonstrating gross-total resection

Upon examination, he had a right temporal visual field cut and right upper extremity drift. He was otherwise neurologically intact. Due to the patient’s new neurologic symptoms and significant mass effect from his left parieto-occipital lesion, he was taken to the operating room for re-resection of the lesion as shown in Fig. 6.

The patient’s drift, headache, and nausea resolved following surgery, but his right temporal visual field cut remained. Postoperative MRI demonstrated gross-total resection of the recurrent lesion. The following month, the patient began hypofractionated Gamma Knife radiosurgery (GKRS) to four targets (left parietal cavity, left anterolateral parietal, right cerebellar, and right frontal), receiving 30 Gy in 3 fractions to each target. The patient is also currently undergoing chemotherapy. An MRI of the brain 6 weeks after his first fraction of GKRS confirmed no new metastases, with decreased sizes of the metastases in the right frontal centrum semiovale and the left lateroparietal lesions.

**Fig. 7:**
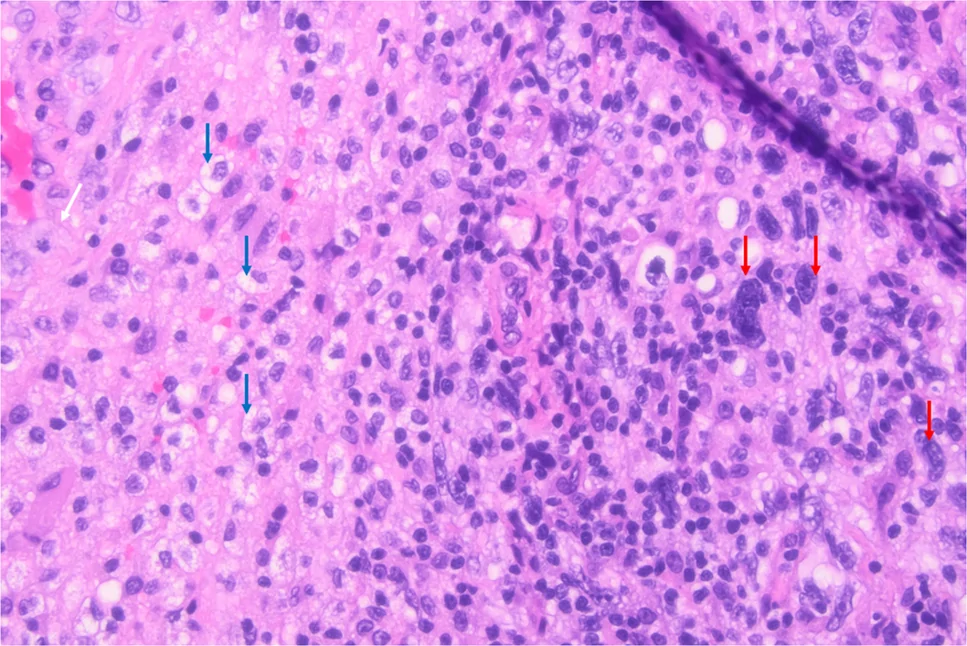
40× view of the re-resected, predominantly cystic left parieto-occipital lesion showing pleomorphism and vacuolated cytoplasm embedded in a fibrillar background

**Fig. 8:**
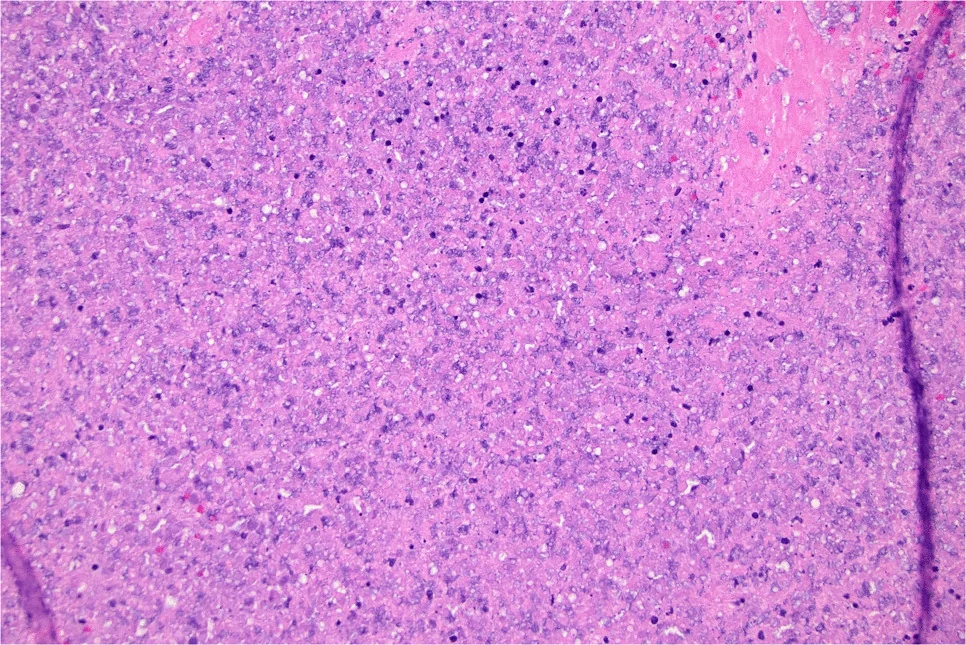
20× view of the re-resected predominantly cystic left parieto-occipital lesion showing necrosis

**Fig. 9:**
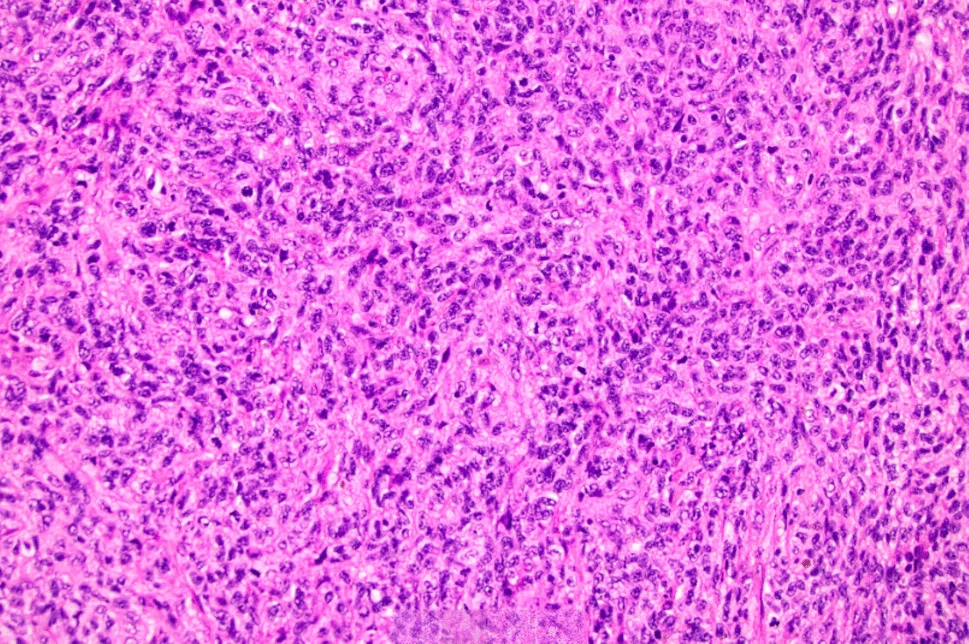
20× view of the re-resected predominantly cystic left parieto-occipital lesion showing pleomorphism and an abundance of mitotic figures

**Fig. 10:**
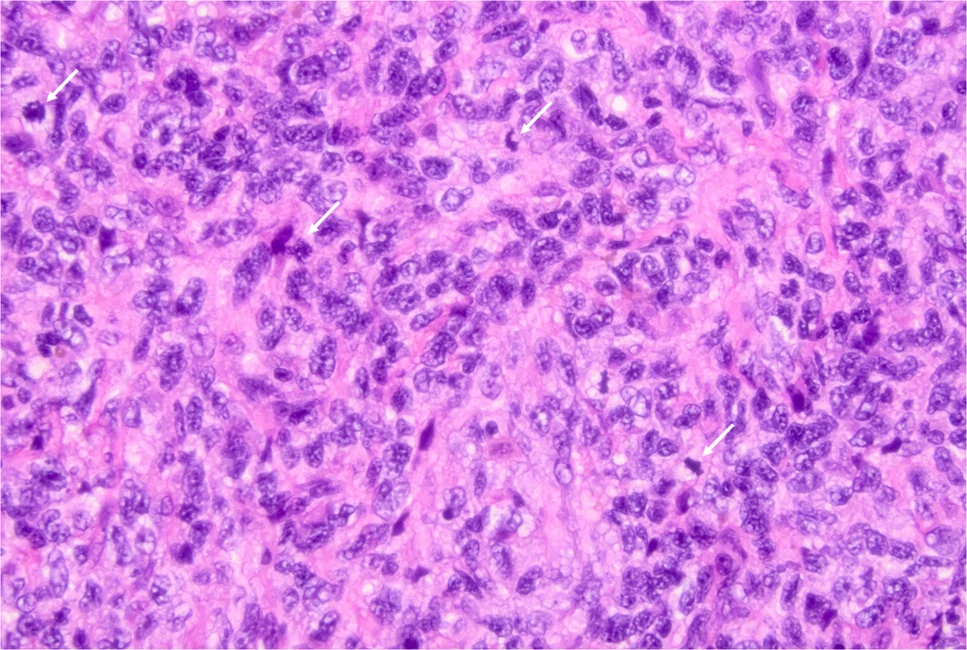
40× view of the re-resected predominantly cystic left parieto-occipital lesion showing pleomorphism and an abundance of mitotic figures

The pathology analysis of re-resected intracranial tissue revealed a densely cellular neoplasm of poorly differentiated cells, ranging from oval to spindle shapes, with pleomorphism, scant cytoplasm, and distinct nucleoli in a fibrillar and sclerotic background. The tumor demonstrated extensive necrosis and numerous mitotic figures with morphology consistent with the previous left parietal resection and left atrial mass excision. Figures 7, 8, 9, and 10 demonstrate the histology from the re-resection, with similar morphological findings with the first resection.

The patient continues to recover as he continues chemotherapy.

## Discussion

Primary cardiac sarcomas are aggressive tumors that often present with metastatic disease and are frequently diagnosed late because symptoms are often nonspecific [5, 6]. In large case series, many patients already have metastatic disease at diagnosis, and median overall survival is roughly a year despite multimodal treatment [4, 5].

Our case aligns with prior reports in that the patient’s tumor originated in the left cardiac atrium and the initial presentation was driven by distant neurologic disease rather than obvious cardiac symptoms [5, 12, 14]. He had multifocal brain lesions at the time of diagnosis along with a large left-sided intracardiac mass, supporting prior reports that left-sided primary cardiac sarcomas have high rates of CNS dissemination [5].

In a 39-patient series, Siontis et al. reported that 12 patients (31%) developed brain metastases including 4 of 18 patients who had metastatic disease at the time of diagnosis [5]. Furthermore, the authors found that brain metastases occurred more often with left-sided tumors which typically developed a median of 8.5 months after sarcoma diagnosis [5]. In a larger cohort of primary malignant cardiac tumors, Guan et al. similarly found frequent metastatic disease and poor long-term survival, with a reported 5-year survival of only 11.5% [4]. These findings align with a systematic review of 19 studies totaling 320 patients with intracranial involvement from primary cardiac tumors, which found that malignant primary tumors were associated with true brain metastases and poorer clinical outcomes compared with benign tumors that more often caused embolic cerebrovascular events [7].

Although only a small number of comparable cases have been published, several reports share important similarities with our patient. Pasalic et al. reported a left atrial sarcoma presenting with progressive neurologic decline due to brain metastases in the absence of preceding cardiac symptoms [12]. Badaloni et al. reported a primary cardiac high-grade myxofibrosarcoma presenting with multiple brain metastases [14]. Lastly, Sun et al. and Marcela et al. each described primary cardiac sarcoma complicated by both embolic cerebrovascular events and brain metastasis, supporting that embolic and metastatic CNS processes can occur simultaneously, as did with our patient [13, 15].

Our patient’s initial MRI scan revealed two enhancing lesions with surrounding vasogenic edema: one later discovered to be a metastasis and the other an embolism from the atrial sarcoma. The radiographic similarities of embolic infarct and tumor can make it difficult to discern appropriate operative targets. The embolic focus could be distinguished from the tumor by its susceptibility-weighted imaging signal and relative reduction in size and edema over a few weeks compared to the tumor’s rapid growth and recurrence following resection.

Despite these similarities, our case differs from prior reports in several ways. The timing of recurrence within weeks in our case was particularly notable, suggesting aggressive sarcoma and supporting the use of close postoperative MRI surveillance with a low threshold for re-intervention when mass effect returns. Age is another distinguishing feature of our case given that most prior cases involve adults [12–16]. Although pediatric and adolescent primary cardiac sarcomas are included in some series, teenage patients presenting with multiple brain lesions and a large left atrial mass remain uncommon in the literature.

Regarding treatment, this case emphasizes the importance of controlling both the intracranial and cardiac disease manifestations. Siontis et al. reported that overall survival was significantly improved in patients who underwent surgical resection of the primary tumor and in those who received chemotherapy, although surgery alone was not sufficient to prevent recurrence or new metastases and relapse was common [5]. Other cases have reported combined approaches, such as craniotomy for symptomatic lesions, adjuvant radiotherapy for residual or multifocal disease, and aggressive surgical management of the cardiac primary tumor when possible [7, 10, 12, 13]. In our patient, brain tumor resection provided relief from mass effect and resection of the left atrial mass was important to address the likely source of emboli and metastatic spread to the CNS.

Notable challenges with our patient included very rapid recurrence of intracranial metastasis requiring multiple surgeries and the need for cardiac surgery. These surgeries often delay initiation of chemotherapy and radiosurgery: the cost of this reality must be carefully considered during treatment planning. Cardiac pathologies secondary to cardiac lesions increased the risk of complications from resection of both the primary and metastatic lesions. Moreover, both the neurosurgical and cardiac procedures had to be completed before chemotherapy could be initiated, which was delayed in part due to the complex pathological testing required to determine a diagnosis.

## Conclusions

In summary, this case illustrates an aggressive, metastatic left atrial cardiac sarcoma in an adolescent patient presenting with intracranial metastasis, embolic infarcts, and rapid intracranial recurrence. This case adds to prior evidence that left-sided primary cardiac sarcomas may carry a high risk of early CNS involvement and require coordinated, multidisciplinary care across specialties.

## Disclaimer

Paper has not been presented at any other journal/conference/meeting.

## Data Availability

No datasets were generated or analysed during the current study.
